# Two-decade trends and factors associated with overweight and obesity among young adults in Nepal

**DOI:** 10.1093/eurpub/ckac131.137

**Published:** 2022-10-25

**Authors:** S Shakya, S Neupane, P Absetz

**Affiliations:** Faculty of Social Sciences, Tampere University, Tampere, Finland

## Abstract

**Background:**

Young adults are vulnerable to obesity due to different life stresses and challenges, leading to risks of cardiovascular and metabolic disorders. Studies on the long-term trends of overweight and obesity, especially among young people in Nepal are scanty. The problems among this productive age group should be checked and prevented at the earliest. The study aimed to assess more than two decades of trends from 1996 to 2019 of overweight and obesity among Nepalese young adults (18-29 years) and the socio-demographic factors associated with it.

**Methods:**

We utilized data from the nationwide Demographic and Health Survey (DHS) and WHO STEPwise approach to surveillance (STEPS) survey. These surveys adopted multistage stratified cluster sampling techniques and used house-to-house structured interviews for data collection. We assessed the prevalence of overweight (BMI 25-29.9 kg/m2) and obesity (BMI≥30 kg/m2) in 1996, 2001, 2006, 2011, 2012, 2016, and 2019 among 18,714 young adults in total, and evaluated the associated socio-demographic factors from the 2016 survey, using logistic regression model.

**Results:**

The preliminary study findings showed that from 1996 to 2019, overweight in women increased from 1.5% to 17.0%, and obesity from 0.1% to 3.4%. For men, overweight rose from 14.4% to 16.6%, and obesity from 1.3% to 2.5% from 2012 to 2019. Higher age was associated with higher odds of overweight and obesity compared to a younger age. Men were less likely to have overweight (AOR: 0.68, CI: 0.53-0.88) and obesity (AOR: 0.42, CI: 0.23-0.78) compared to women. Moreover, urban residents had higher odds of having obesity (AOR: 2.35, CI: 1.25-4.44) compared to rural residents.

**Conclusions:**

Overweight and obesity have rising trends among young adults in Nepal. Older age, female and urban residence were associated with higher odds of overweight and obesity. Therefore, the interventions targeted to the risk groups can help in curbing the increasing obesity trends in Nepal.

**Key messages:**

• The information on trends and the factors associated with overweight and obesity may help to identify needs and opportunities to halt the rising obesity trend and prevent risk factors.

• The study findings can guide in formulating a national strategy to combat overweight and obesity among Nepalese youths.

